# Attenuated Levels of Hippocampal Connexin 43 and its Phosphorylation Correlate with Antidepressant- and Anxiolytic-Like Activities in Mice

**DOI:** 10.3389/fncel.2015.00490

**Published:** 2015-12-22

**Authors:** Gaël Quesseveur, Benjamin Portal, Jean-Arnaud Basile, Pascal Ezan, Alexia Mathou, Hélène Halley, Corinne Leloup, Xavier Fioramonti, Nicole Déglon, Christian Giaume, Claire Rampon, Bruno P. Guiard

**Affiliations:** ^1^Institut National de la Santé et de la Recherche Médicale UMR-S 1178 – Dépression, Plasticité and Résistance Aux Antidépresseurs, Laboratoire de Neuropharmacologie EA 3544, Faculté de Pharmacie, Université Paris-SudChâtenay-Malabry, France; ^2^Centre National de la Recherche Scientifique, Centre de Recherches sur la Cognition Animale UMR 5169, Centre de Biologie Intégrative, Université Toulouse III– Paul SabatierToulouse, France; ^3^Center for Interdisciplinary Research in Biology, Centre National de la Recherche Scientifique UMR 7241, Collège de FranceParis, France; ^4^Centre des Sciences du Goût et de l’Alimentation – Centre National de la Recherche Scientifique UMR 6265 – Institut National de la Recherche Agronomique UMR 1324, Université de BourgogneDijon, France; ^5^Laboratory of Cellular and Molecular Neurotherapies, Department of Clinical Neurosciences, Centre Hospitalier Universitaire VaudoisLausanne, Switzerland

**Keywords:** astrocytes, connexin 43, antidepressants, depression, anxiety, hippocampus, stress, behavior

## Abstract

Clinical and preclinical studies have implicated glial anomalies in major depression. Conversely, evidence suggests that the activity of antidepressant drugs is based, at least in part, on their ability to stimulate density and/or activity of astrocytes, a major glial cell population. Despite this recent evidence, little is known about the mechanism(s) by which astrocytes regulate emotionality. Glial cells communicate with each other through gap junction channels (GJCs), while they can also directly interact with neurons by releasing gliotransmitters in the extracellular compartment via an hemichannels (HCs)-dependent process. Both GJCs and HCs are formed by two main protein subunits: connexins (Cx) 30 and 43 (Cx30 and Cx43). Here we investigate the role of hippocampal Cx43 in the regulation of depression-like symptoms using genetic and pharmacological approaches. The first aim of this study was to evaluate the impact of the constitutive knock-down of Cx43 on a set of behaviors known to be affected in depression. Conversely, the expression of Cx43 was assessed in the hippocampus of mice subjected to prolonged corticosterone (CORT) exposure, given either alone or in combination with an antidepressant drug, the selective serotonin reuptake inhibitor fluoxetine. Our results indicate that the constitutive deficiency of Cx43 resulted in the expression of some characteristic hallmarks of antidepressant-/anxiolytic-like behavioral activities along with an improvement of cognitive performances. Moreover, in a new cohort of wild-type mice, we showed that CORT exposure elicited anxiety and depression-like abnormalities that were reversed by chronic administration of fluoxetine. Remarkably, CORT also increased hippocampal amounts of phosphorylated form of Cx43 whereas fluoxetine treatment normalized this parameter. From these results, we envision that antidepressant drugs may exert their therapeutic activity by decreasing the expression and/or activity of Cx43 resulting from a lower level of phosphorylation in the hippocampus.

## Introduction

Major depression (MD) is one of the most common mental disorders, affecting 350 million people worldwide and is a leading cause of disability (Milanovic et al., 2015). MD imposes substantial economic costs and its impact on disability, productivity, and quality of life further accentuates these costs (Lecrubier, 2001). Evidence indicates that stressful life events increase the risk for MD (Binder and Nemeroff, 2010). As a consequence, classically used animal models of depression rely on the activation of the hypothalamus-pituitary-adrenal (HPA) axis in order to reproduce part of the physiological, neurochemical and morphological maladaptive changes detected in depressed patients (Dalvi and Lucki, 1999). Selective serotonin reuptake inhibitors (SSRIs) represent the most widely prescribed class of antidepressant drugs, but approximately 50% of depressed patients do not respond adequately to SSRI. Moreover even in responders, the onset of therapeutic action is of 2–4 weeks (Blier, 2001). Considering the prevalence of MD and its consequences on life’s quality, a better knowledge of its pathophysiology is required to envision the identification of new therapeutic targets.

Opening the way for a promising area of pharmacological interventions, recent data suggest that glia plays an important role in MD and in antidepressant drugs response (Quesseveur et al., 2013a). Indeed, reduced expression of the glial fibrillary acidic protein (GFAP) has been observed in the brain of depressed patients (Miguel-Hidalgo et al., 2000; Webster et al., 2001; Si et al., 2004; Nagy et al., 2015). This glial anomaly, specifically identified in limbic regions (Torres-Platas et al., 2015), is not restricted to GFAP since a decrease in cortical mRNA expression of astrocyte-enriched genes including ALDH1L1, SOX9, GLUL, SCL1A3 has been reported in a large cohort of depressed patients (Nagy et al., 2015). In agreement with the latter findings, subjecting animals to unpredictable chronic mild stress (UCMS) or prolonged social defeat, two animal models of depression, also elicits an attenuation of GFAP (mRNA or protein) in the cortex (Banasr et al., 2010) or the hippocampus of rats (Czeh et al., 2006; Araya-Callis et al., 2012). An elevation in stress-induced glucocorticoids might have contributed to this effect as studies indicated that corticosterone (CORT) can lower GFAP levels in the rat brain (Nichols et al., 1990). Despite this evidence, little is known about the mechanism(s) by which astrocytic activity may regulate emotionality and its related brain circuits.

Astrocytes have the ability to communicate with each other through GJCs, while they can also communicate directly with neurons by releasing gliotransmitters in the extracellular compartment via hemichannels (HCs). In astrocytes, both GJCs and HCs are formed by two main subunits: connexins 30 and 43 (Cx30 and Cx43; Rouach and Giaume, 2001). A few months ago, a post-mortem study correlated MD with decreased levels of Cx43 immunoreactivity in the prefrontal cortex (Miguel-Hidalgo et al., 2014). Similarly, rats subjected to UCMS exhibit a significantly decreased expression and function of cortical and hippocampal Cx43 GJCs whereas typical antidepressant drugs such as fluoxetine, duloxetine, or the tricyclic amitriptyline increased astroglial Cx43 protein levels (Li et al., 2010; Sun et al., 2012; Morioka et al., 2014; Mostafavi et al., 2014). Although the latter studies focused their attention on Cx43 without providing information about Cx30, these clinical and preclinical observations raise the possibility that the down-regulation and/or functional inactivation of astroglial Cx GJCs could produce deleterious effects on mood. Conversely, the over-expression and/or activation of astroglial Cxs GJCs might underlie, at least in part, antidepressant drugs response. However, the role of astroglial Cxs in the regulation of emotional states could be more complicated than expected. Indeed, it was recently reported that restraint stress increases the opening of Cx43 HCs in the hippocampus, thereby promoting the release of glutamate in a neurotoxic concentration for neighboring cells (Orellana et al., 2015). Connexin 43 HCs activity may thus constitute a pre-requisite condition for the damaging effects of chronic stress on neuronal activity and related functions. Consequently and given the importance of adult hippocampal neurogenesis in antidepressant drug response (Santarelli et al., 2001; Surget et al., 2008; David et al., 2009), the latter results suggest, that the inactivation of Cx43 HCs might positively influence emotionality.

Here, we questioned the role of hippocampal Cx43 in the regulation of emotion-related behaviors using complementary genetic and pharmacological approaches. In the first part of this study, we evaluated the impact of the constitutive knock-down of Cx43 on a set of well-defined behavioral paradigms known to recapitulate several symptoms of depression. The second part of this work was designed to investigate the putative changes in hippocampal Cx43 expression in mice chronically exposed to the stress hormone CORT, either alone or in combination with the SSRI fluoxetine. The choice of the hippocampus was based on the fact that this brain region is subjected to intense structural changes and remodeling in MD while an important part of the therapeutic activity of antidepressant drugs relies on their ability to block or reverse impairments of plasticity caused by stress or related pathologies (Warner-Schmidt and Duman, 2006).

## Materials and Methods

### Animals

For experiment 1, 7 week-old male transgenic Cx43^fl/fl^ mice were used and wild-type littermates (Cx43^wt/wt^) with a similar mixed 129P2-C57BL/6 background served as controls (Theis et al., 2001). For experiments 2 and 3, 7 week-old wild-type C57BL/6J mice were used. All mice weighed between 25 and 35 g at the time of behavioral assessment. Mice were housed by 4–5 per cage under standard conditions (12:12 h light-dark cycle, light on at 7 am, 22 ± 1°C ambient temperature, 60% relative humidity). The “Centre de Recherches sur la Cognition Animale (CRCA)” has received French legal approval for experiments on living vertebrate animals (Arrêté Préfectoral: 9-02-2011). This work was carried out in accordance with the Policies of the French Committee of Ethics. Animal surgery and experimentations conducted in this study were authorized by the French Direction of Veterinary Services to CR (#31-11555521, 2002) and all efforts were made to improve animals’ welfare and minimize animals suffering.

### Drugs and Surgery

Corticosterone (from Sigma–Aldrich, Saint-Quentin Fallavier, France) was dissolved in vehicle (beta-cyclodextrin 0.45%) as previously described (David et al., 2009). CORT (35 μg/ml, equivalent 5 mg/kg/day) was delivered alone or in presence of the antidepressant drug fluoxetine in opaque bottles to protect it from light, available *ad libitum* in the drinking water. Fluoxetine hydrochloride (18 mg/kg per day for 1 month) was purchased from Anawa Trading (Wangen, Switzerland) and was used at a dose that reversed CORT-induced behavioral anomalies (David et al., 2009). In this study, we also used the non-selective Cx blocker carbenoxolone (CBX; Rouach et al., 2003) at a concentration of 10 mM based on a previous report showing its ability to modulate memory (Bissiere et al., 2011; Sun et al., 2012). One week before the tests, wild-type mice were anesthetized with chloral hydrate (400 mg/kg; i.p.). They were then placed on a stereotaxic frame and bilaterally implanted with 29 gage guide cannulae (Cooper’s needle works LTD) in the ventral hippocampus using the following coordinates: -2.5 posterior from bregma, ±2.8 mm lateral to midline and 2.0 ventral from the dura. The implants were secured to the skull with dental acrylic. Dummy cannulae were inserted from the end of surgery until the infusion day to prevent clogging of the guides. For its bilateral micro-infusion in the ventral hippocampus (0.3 μl per side at a constant rate of 0.1 μl/min), CBX was dissolved in 0.1 M sterile PBS. 33 gage internal injection cannulae, extending 0.5 mm beyond the guide cannulae, were inserted and connected to a microsyringe driven by a microinfusion pump. The cannulae were left in place for an additional 2 min before being withdrawn and the animals were allowed an additional 15 min before the beginning of experimentation. Vehicle animals were also cannulated and received similar volumes of 0.1 M sterile PBS.

### Behavioral Tests

All behavioral tests were performed in the morning to avoid differences in locomotor activity and other variables affected by circadian rhythm. Previous studies have indicated that certain test variables are sensitive, whereas others are resistant, to test order (McIlwain et al., 2001). Bearing this in mind, performance was then evaluated from the least to the most stressful test, thereby decreasing the chance that one test might impact the behavior evaluated in the subsequent paradigm. Importantly, since prior handling and testing have been described to reduce exploratory activity and emotionality in mice (Võikar et al., 2004), animals were tested only once in each paradigm. Finally, given that it has been demonstrated that the interval between behavioral tests could be as short as 1 day, with a weak effect on overall performance (Paylor et al., 2006), in the present study, a 2-day recovery period between tests was provided. It is also important to state that by reducing the inter-test interval, we reduced the possible effects of time on drug administration on tests.

***The Sucrose preference test (SPT)*** was performed in singled-house mice habituated for 48 h to drink water from two bottles. On the following 3 days, the mice could choose between a water bottle and a 1% sucrose solution bottle, switched daily. Sucrose solution intake for 24 h was measured during the last day and expressed as a percentage of the total amount of liquid ingested and normalized to the mouse body weight.

***The splash test (ST)*** was performed for a 5-min period as previously described (David et al., 2009). After squirting 200 μl of a 10% sucrose solution on the mouse’s snout, grooming time was scored by a single experimenter as an index of self-care.

***The tail suspension test (TST)*** was performed using the BIOSEB’s Tail Suspension Test system (Bioseb) during a 6-min session period. Immobility time was scored as an index of resignation. Movements in terms of energy and power in motion were measured to ensure the absence of any locomotor bias.

***The Elevated plus maze (EPM)*** was performed by placing animals into the central area facing one closed arm and were allowed to explore the maze for 5 min. Testing took place in bright dimmed light conditions (800–900 lux). Automated scoring was done using ANY-maze(tm) behavioral video-tracking software from Stoelting Co (Bioseb, Vitrolles, France). Total number of entries and time spent in the open arms were measured.

***The Open field (OF)*** was performed in 40 cm × 40 cm Plexiglas boxes (Mouse Open Field Arena ENV-510; Med Associates Inc.) during a 30-min session period. Activity chambers were computer interfaced for data analysis (SOF-811; Med associates Inc.), and two regions were defined by grid lines that divided each box into center and periphery areas, with each of the four lines located 11 cm away from each wall. The total number of entries, the ambulatory distance and the time spent in each compartment were measured.

***The Novelty suppressed feeding (NSF)*** was performed in a white plastic box (30 cm × 60 cm). Mice were food deprived for 24 h before testing and then placed in the corner of the box with their respective food pellet on a white square filter paper at the center of the arena under a bright light (∼60 W) placed about 60–80 cm above the food pellet. Latency to begin eating was scored by a single experimenter, with a cut-off time of 10 min. Upon return to their home-cage, the total amount of food intake was measured for each animal and for a 5-min period to ensure the absence of differences in hunger/motivation to eat.

***The Object location test (OLT)* and *the Object recognition test (ORT)*** started with an habituation to the open-field by exposing the mice to the apparatus for 10 min 1 day before starting the trials (day 1). During the training session, two identical objects were placed 15 cm away from the two opposite corners (day 2). Each mouse was placed in the center of the open-field, facing the objects and was allowed to freely explore the objects for 10 min. During the retention session held 24 h later (day 3), one object was displaced to a new position (in the OLT), or replaced by another one (in the ORT). Then, each mouse was placed in the center of the open-field facing the objects and was allowed to freely move for 10 min. Time spent exploring the different objects was recorded using Noldus Ethovision software.

***The Fear conditioning (FC)*** consisted of a single conditioning session. During conditioning, each mouse was introduced into the conditioning chamber for a total of 4 min 30 s. After a 2-min exploration period, a sound (CS) was emitted for 30 s, and a foot-shock (US) was superposed to the tone during the last 2 s. The mice were then maintained in the chamber for two additional min. After this procedure, the mouse was gently removed from the chamber. 24 h after the conditioning session, freezing to the context was assessed by again placing each mouse in the conditioning chamber. The level of freezing was measured during 4 min, no tone or foot-shock being presented to the animal. Two hours later, mice were tested for freezing to the tone in a modified context. Two minutes after introduction in the modified chamber, freezing was recorded during a 2-min tone presentation. Freezing behavior was both automatically recorded and scored by an experimenter blind to the experimental conditions. Data was calculated for the 4-min context recall test, the 2-min pre-tone, and the 2-min tone tests.

### Emotionality *z*-Score

*Z*-normalization across complementary measures of emotionality-related behaviors assessed from different paradigms was applied after each experimental protocol. Simple mathematical tools were used to normalize data from each individual raw behavioral data to the mean of the control groups within each experimental cohort. Data were then integrated into a single value, named emotionality *z*-scores. Values were obtained by subtracting the average of observations in a population from an individual raw value and then dividing this difference by the population standard deviation as described previously (Guilloux et al., 2011; Petit et al., 2014). This type of normalization allows data on different scales to be compared. The emotionality *z*-score included the parameters measured in the ST (grooming time), the TST (immobility time), the elevated plus maze (open arms entries and open arms times), the open-field (center entries, center time, and center to total distance ratio) and the NSF (latency to feed). It is noteworthy that several parameters were calculated in the OF and EPM to evaluate anxiety. In order to have a same impact of each test on the emotionality *z*-score, we averaged the normalized parameters evaluated in the OF and EPM to obtain a single value per mouse and per behavioral test. More details about the mathematical method for emotionality *z*-score calculation are provided in Supplementary Table S1.

### Western-Blot

Expression of Cx30 and Cx43 were analyzed by western blot as previously described (Ezan et al., 2012). Briefly, frozen hippocampi or hypothalamus were pulverized on dry ice, resuspended in boiling 2% SDS containing protease inhibitors (Roche), β-glycerophosphate (10 mM) and orthovanadate (1 mM), and sonicated on ice. Lysates were centrifugated at 13,000 rpm at 4°C and supernatants were diluted with 5x Laemmli buffer, and boiled 5 min. After quantification using the BCA protein assay kit (Pierce), protein samples (20 μg/lane) were separated by electrophoresis on 10% polyacrylamide gels (Gel Nupage 4–12%, Bis-Tris) and transferred onto nitrocellulose membranes. Membranes were blocked for 1 h with 5% fat-free dried milk/TBS1x-0.1% Tween and incubated overnight at 4°C either with anti-Cx30 (rabbit polyclonal, Invitrogen 71-2200, 1/500) or anti-Cx43 (mouse monoclonal, BD Biosciences 610062, 1/500). The next day membranes were washed and incubated with peroxydase-conjugated goat anti-rabbit or goat anti-mouse (HRP-IgG, Santa Cruz, respectively, sc-2004 or sc-2005, 1/2500), for 2 h at room temperature. Chemiluminescent signal was obtained using Western Lighting Plus ECL detection kit (Perkin-Elmer NEL104001EA). Blots were reprobed with anti-tubulin or anti-actin (mouse monoclonal, Sigma, 1/10,000 and 1/30,000; respectively, T6199, A3853) to check protein load. Semi-quantitative densitometric analysis was performed using ImageJ software. The analysis of Cx43 activity was assessed through its level of phosphorylation.

### Experimental Design

In the experiment 1 (Supplementary Figure S1A), control Cx43^wt/wt^ and Cx43^fl/fl^ mice were subjected to a full comprehensive battery of behavioral tests to evaluate their phenotype. Separate cohorts were used to assess putative changes in depressive, anxious and cognitive symptoms.

In the experiment 2 (Supplementary Figure S1B), wild-type mice were subjected to CORT or its vehicle for 1 month and then administered with vehicle or fluoxetine for one additional month. The efficacy of CORT or antidepressants on behavioral performances were assessed in different paradigms. At the end of this procedure, mice brains were removed for the analysis of Cx43 activity through its level of phosphorylation.

### Statistical Analysis

For all experiments, statistical data analysis (StatView 5.0 Abacus Concepts, Berkeley, CA, USA) used means ± SEM. Student *t*-tests and one-way ANOVA followed on treatment factor by Fisher Protected Least Significance Difference *post hoc* test were used. The linear relationship between emotionality *z*-scores and the level of expression of non-phosphorylated Cx43 was analyzed by the Pearson’s correlation coefficient after a Shapiro-Wik normality test. Significant level was set at *p* < 0.05.

## Results

### Cx43 Controls Behavioral Symptoms Related to Depression and Anxiety

**FIGURE 1:**
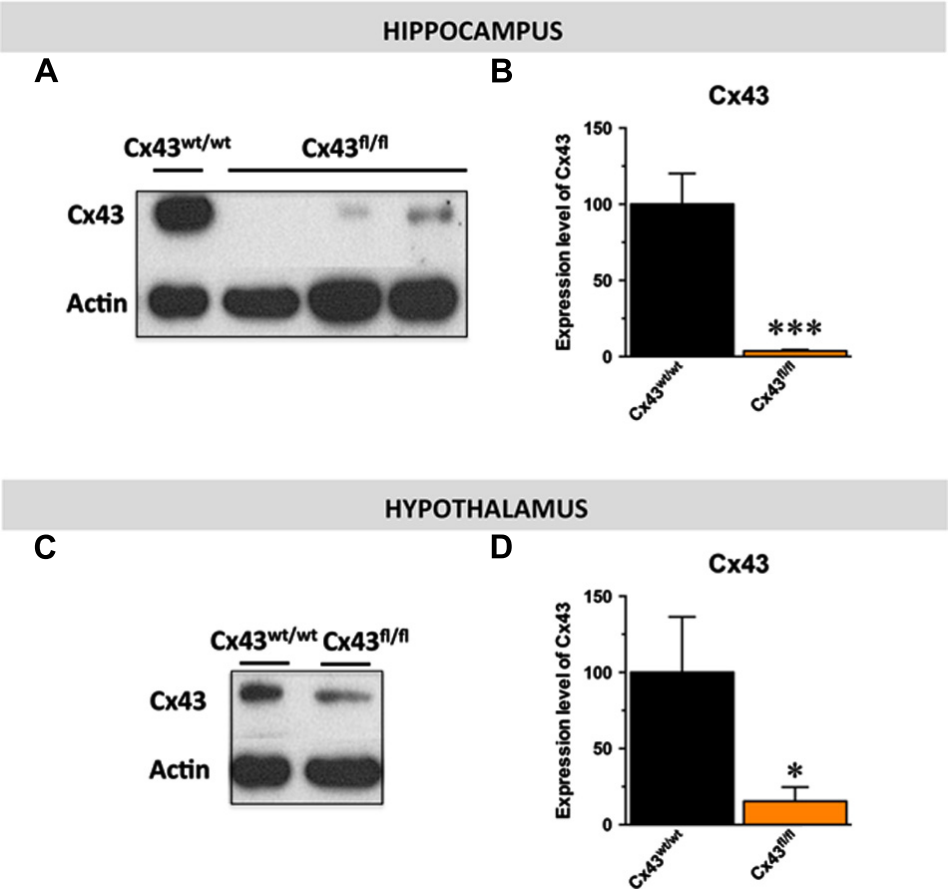
**Expression of astroglial Cx43 in Cx43^fl/fl^ mice.** Immunoblot analysis **(A–C)** and quantification **(B–D)** of Cx43 levels in the hippocampus **(B)** and the hypothalamus **(D)** of Cx43^fl/fl^ mice relative to control Cx43^wt/wt^ mice. β-actin was used as a loading control. ^∗^*p* < 0.05 and ^∗∗∗^*p* < 0.001: significantly different from Cx43^wt/wt^ (*n* = 6–7 mice/group for the hippocampus et *n* = 4 for the hypothalamus).

Given the distribution of Cx43 in astroglial and stem cells in the central nervous system (Dermietzel et al., 1989; Kunze et al., 2009), we proposed to generate mice with a specific inactivation of astroglial Cx43 in the hippocampus. To this end, we generated a pseudotyped MOKOLA lentiviral vectors to selectively drive the *in vivo* expression of the Cre-recombinase within hippocampal astrocytes of Cx43^fl/fl^ mice. The envelope confers to the virus a tropism toward these glial cells (Colin et al., 2009; Quesseveur et al., 2013b) and coupling with a detargeting strategy using miRNA9T and miRNA124T eliminates the putative residual expression of this Cre-recombinase in hippocampal stem cells and neurons, respectively, thus providing a selective astrocytic targeting. However, and as previously shown (Theis et al., 2003; Unger et al., 2012), we observed that Cx43^fl/fl^ mice themselves displayed a dramatic decrease in the expression of Cx43 relative to control Cx43^wt/wt^ mice in various brain regions including the hippocampus (∼-95%; *t*_1,11_ < 0.001) and the hypothalamus (∼-85%; *t*_1,6_ < 0.05; **Figures 1A–D**). On this background, the relevance and the interest of our strategy consisting in over-expressing the Cre-recombinase within hippocampal astrocytes was limited. However, we took advantage of the knock-down of this protein to assess the consequences of constitutive Cx43 deficiency in a set of behavioral aspects known to be affected in depression: hedonic state, behavioral despair, self-care, anxiety, and cognition/memory by comparing Cx43^fl/fl^ mice and Cx43^wt/wt^ mice maintained on the same genetic background.

**FIGURE 2:**
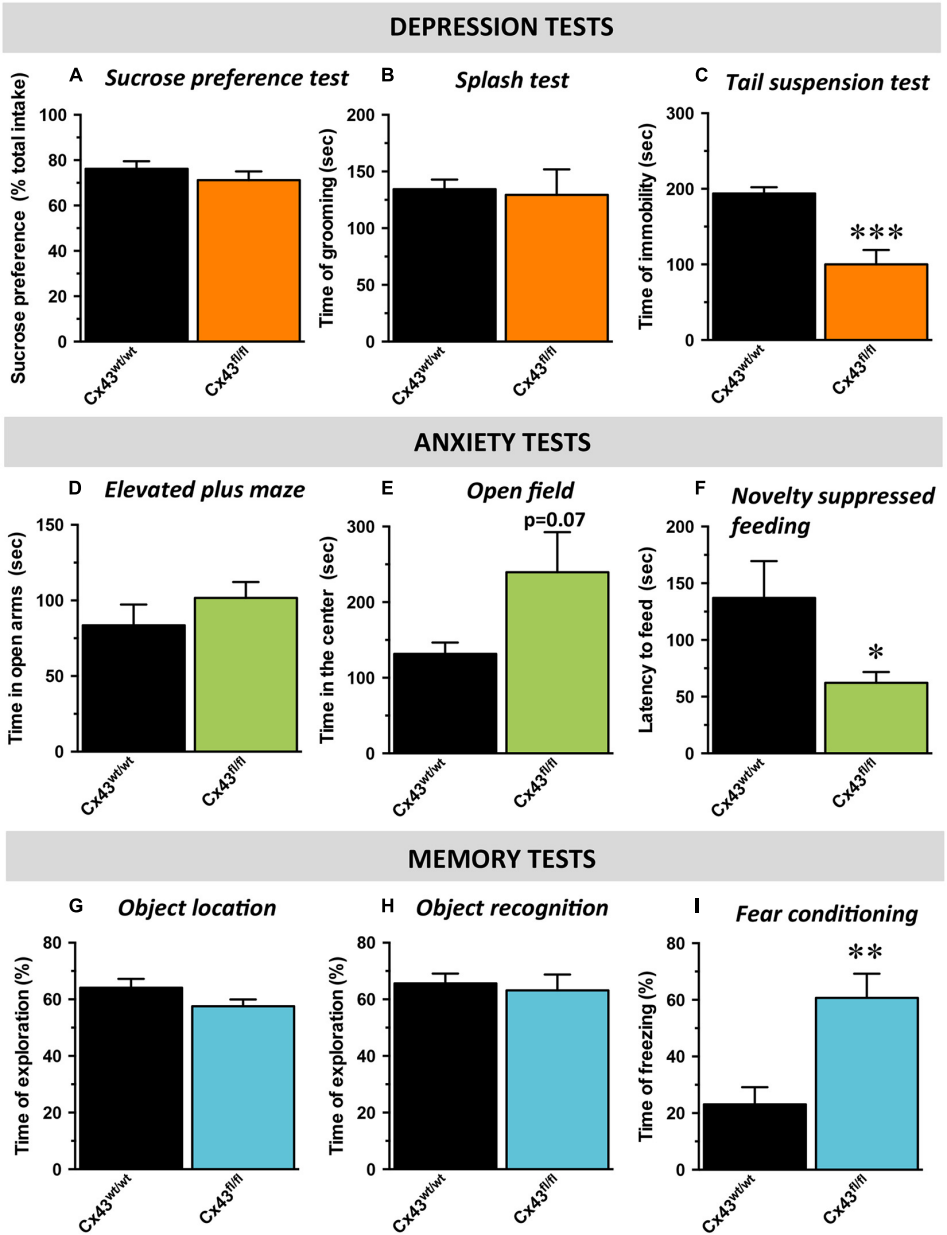
**Behavioral phenotype of Cx43^fl/fl^ mice related to mood, anxiety, and memory.** Data are expressed as mean ± SEM. **(A–C)** Mood evaluated by the sucrose consumption in the sucrose preference test (SPT; **A)**, the time of grooming in the splash test (ST, **B**) and the time of immobility in the tail suspension test (TST; **C**). **(D,E)** Anxiety evaluated by the time in the open arms of the elevated plus maze (EPM; **D**), the time in the center of the open field (OF; **E**) and the latency to feed in the novelty suppressed feeding (NSF; **F**). **(G–I)** cognitive performances evaluated by the time of exploration of the displaced object in the object location test (OL; **G**), of exploration of the novel object in the object recognition test (OR; **H**) and the time of freezing in the contextual fear conditioning (CFC; **I**). ^∗^*p* < 0.05, ^∗∗^*p* < 0.01, ^∗∗∗^*p* < 0.001: significantly different from Cx43^wt/wt^ (*n* = 8–10 mice/group).

With respect to hedonic state, no differences in sucrose preference was detected between control Cx43^wt/wt^ and Cx43^fl/fl^ mice (*t*_1,16_ = 0.34; **Figure 2A**) suggesting that the loss of Cx43 did not interfere with mice’s ability to experience pleasure. In the ST evaluating self-care, no difference in the time of grooming was noticed between Cx43^wt/wt^ and Cx43^fl/fl^ mice (*t*_1,16_ = 0.8; **Figure 2B**). We also assessed immobility during exposure to inescapable stress using the TST. Cx43^fl/fl^ mice showed a marked decrease in the immobility time compared to Cx43^wt/wt^ mice, indicative of a low level of despair (*t*_1,16_ < 0.001; **Figure 2C**).

Mice were then tested in behavioral paradigms assessing anxiety. In the elevated plus maze, no difference was observed between Cx43^wt/wt^ and Cx43^fl/fl^ mice in the time spent in the open arms (*t*_1,15_ = 0.3; **Figure 2D**) nor in the number of entries in these arms (*t*_1,15_ = 0.4, data not shown). In the OF, which evaluates locomotor activity in a novel environment as well as the level of anxiety, an increase, albeit not statistically significant, in the time spent (*t*_1,12_ = 0.07; **Figure 2E**) and the number of entries in the center area (*t*_1,12_ = 0.09; data not shown) was observed in Cx43^fl/fl^ mice. To eliminate a putative bias, we verified that the loss of Cx43 did not affect the total distance traveled (Cx43^wt/wt^: 8595 ± 575 vs. Cx43^fl/fl^: 7679 ± 598 cm; *t*_1,12_ = 0.3). In the NSF, which evaluates animal’s latency to consume food in a novel aversive environment, Cx43^fl/fl^ mice exhibited a decreased latency to feed compared to Cx43^wt/wt^ mice (*t*_1,16_ < 0.05; **Figure 2F** and Supplementary Figure S2A for survival curve). Importantly, body weight loss and food consumption in the home-cage were similar in both genotypes (Supplementary Figures S2B,C).

Finally, cognitive/memory functions were assessed by the measure of performances in the OLT (spatial memory), in the ORT (non-spatial working memory) and in the fear conditioning (contextual memory) successively. In the OLT, we found that Cx43^fl/fl^ mice did not displayed notable spatial memory deficits compared to control Cx43^wt/wt^ mice. Indeed, during memory testing 24 h after the acquisition phase, exploratory preference for mice of both groups were significantly higher than chance value (50%), indicating that animals from both genotypes remembered the initial position of the objects (Cx43^wt/wt^ mice: *p* < 0.01 and Cx43^fl/fl^ mice: *p* < 0.05; **Figure 2G**). Moreover, Cx43^fl/fl^ and control Cx43^wt/wt^ mice similarly explored the displaced object (*t*_1,15_ = 0.1; **Figure 2G**) indicating that Cx43 know-down had no effect on spatial memory in this task.

During memory testing in the ORT, Cx43^fl/fl^ and control Cx43^wt/wt^ mice also explored to the same extent the novel object (*t*_1,15_ = 0.6) and both values were significantly different from chance value (Cx43^wt/wt^ mice: *p* < 0.05 and Cx43^fl/fl^ mice: *p* < 0.01; **Figure 2H**). It is noteworthy that during the acquisition phase of object location (*p* = 0.5) and ORT (*p* = 0.7), both groups of mice spent the same amount of time exploring the objects (Supplementary Figures S2D,E). In the fear conditioning, 24 h after the conditioning session, Cx43^fl/fl^ mice exhibited significantly more freezing than Cx43^wt/wt^ mice (*t*_1,15_ < 0.01; **Figure 2I**). These data are in favor of an improvement of the associative memory in Cx43^fl/fl^ mice. Interestingly, freezing to the tone was also studied in a modified context. As a validation of the experiment, freezing was measured before the shock on day 1 and no differences occurred between Cx43^fl/fl^ and control Cx43^wt/wt^ mice (*t*_1,15_ = 0.12; Supplementary Figure S2F).

These results indicate that the genetic loss of Cx43 elicits a behavioral phenotype which is similar to that observed in response to the chronic administration of antidepressant drugs.

### Stress and the Selective Serotonin Reuptake Inhibitor Fluoxetine Display Opposite Effects on the Functional Activity of Hippocampal Cx43

**FIGURE 3:**
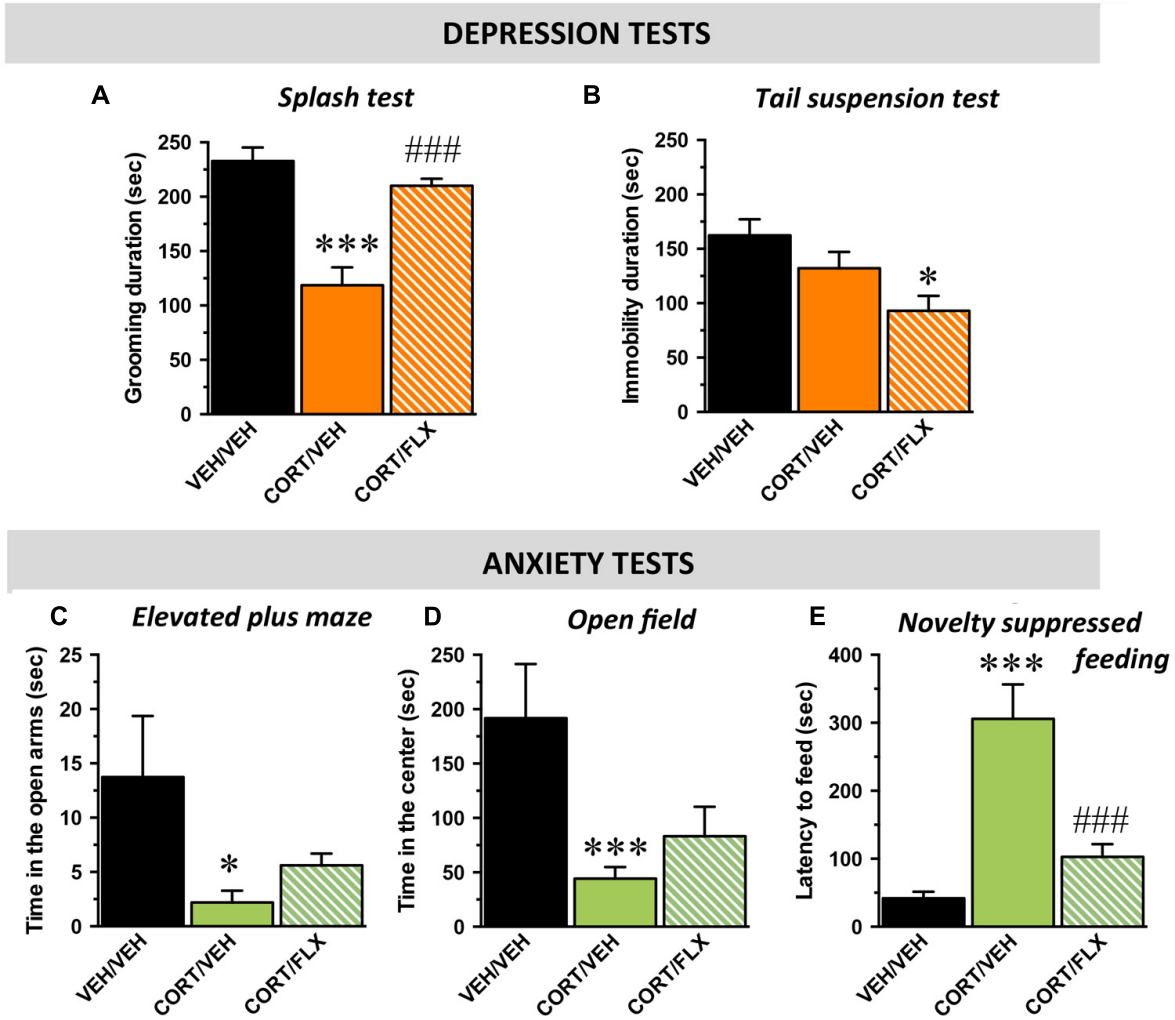
**Behavioral phenotype of wild-type mice subjected to corticosterone (CORT) given alone or in combination with fluoxetine.** Data are expressed as mean ± SEM in wild-type mice administered with with vehicle (VEH) or CORT(35 μg/ml/day; p.o.) given alone or in combination with fluoxetine (FLX; 18 mg/kg/day; p.o.). **(A,B)** Mood evaluated by the time of grooming in the ST **(A)** and the time of immobility in the TST **(B)**. **(C–E)** Anxiety evaluated by the time in the open arms in the elevated plus maze (EPM; **C**), the time in the center of the arena in the **(D)** and the latency to feed in the **(E)**. ^∗^*p* < 0.05, ^∗∗∗^*p* < 0.001: significantly different from the VEH-treated group^.^ ###*p* < 0.001: significantly different from the CORT/VEH-treated group. (*n* = 10–11 mice/group).

Wild-type mice were first tested in behavioral paradigms assessing mood. In the splash and tail suspension tests, the one-way ANOVA indicated a significant effect of treatment factor [*F*_(2,29)_ = 21,4; *p* < 0.001 and *F*_(2,29)_ = 3,7; *p* < 0.05; respectively]. In the ST, chronic CORT exposure induced a significant decrease in the time of grooming relative to control mice (*p* < 0.001) whereas this depressive-like phenotype was reversed by the chronic administration of fluoxetine (18 mg/kg/day for 1 month; p.o.; *p* < 0.001; **Figure 3A**). In the TST, although CORT failed to decrease the time of immobility (*p* > 0.05), the addition of fluoxetine significantly reduced this parameter pointing its antidepressant-like activity (*p* < 0.05; **Figure 3B**).

With respect to anxiety, in the elevated plus maze, the one-way ANOVA on the time spent in the open arms revealed a significant effect of treatment factor [*F*_(2,29)_ = 4,3; *p* < 0.05]. Indeed, CORT decreased this parameter (*p* < 0.05) whereas fluoxetine did not reverse this effect (*p* = 0.3; **Figure 3C**). In the OF, the one-way ANOVA revealed a significant effect of treatment factor on the time spent in the center but not on the number of entries in this compartment [*F*_(2,29)_ = 8,4; *p* < 0.01 and *F*_(2,29)_ = 2,1; *p* = 0.14; respectively]. Hence, CORT decreased the time in the center (*p* < 0.001) and again, fluoxetine failed to significantly reverse this effect (*p* = 0.3; **Figure 3D**). Finally, in the NSF, the one way-ANOVA showed a significant effect of treatment factor [*F*_(2,29)_ = 14,6; *p* < 0.001]. CORT induced a significant increase in the latency to feed (*p* < 0.001) and this effect was reversed by fluoxetine (*p* < 0.001; **Figure 3E**). To eliminate putative bias, body weight loss and food consumption in the home cage were monitored and no differences were observed between the experimental groups [*F*_(2,29)_ = 0,1; *p* = 0.8 and *F*_(2,29)_ = 0,2; *p* = 0.7; respectively] (Supplementary Figures S3A–C).

**FIGURE 4:**
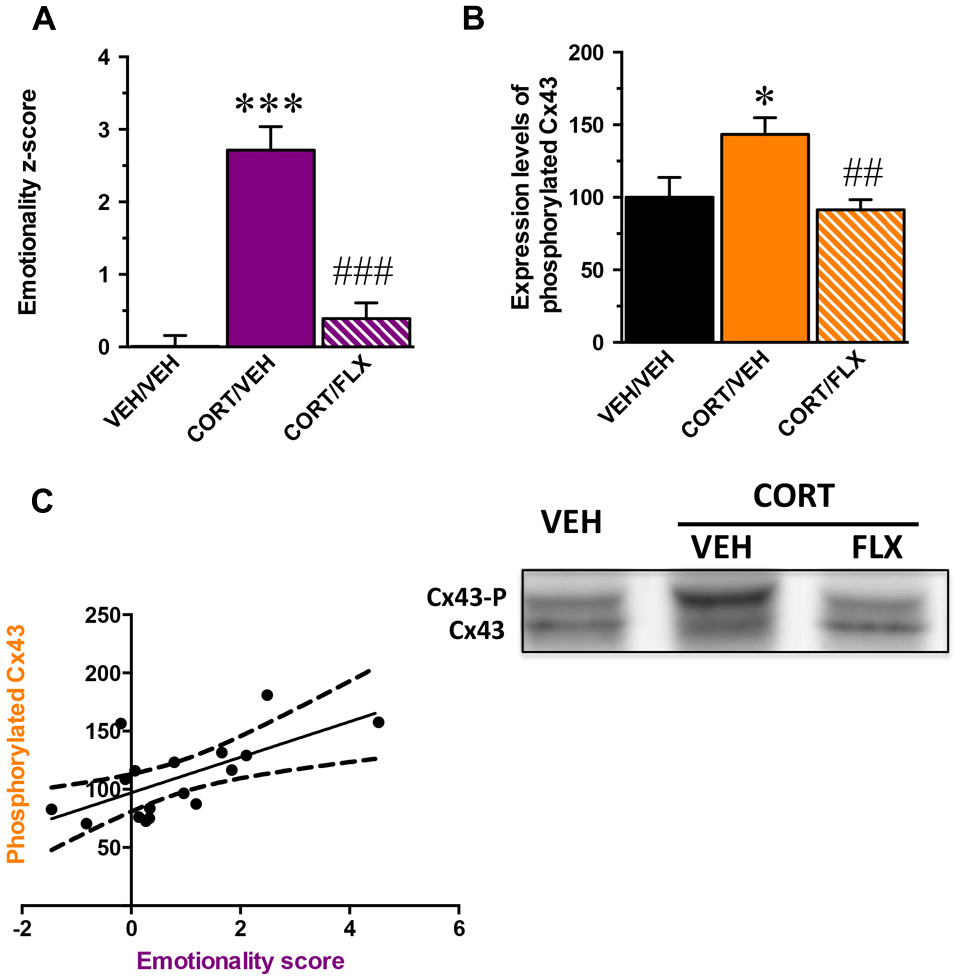
**Correlation between the phosphorylation state of Connexin 43 and the emotionality-score. (A)** emotionality *z*-score integrating all behavioral parameters assessed in the ST, test suspension test, elevated plus maze, OF, and NSF in mice administered with vehicle (VEH) or CORT(35 μg/ml/day; p.o.) given alone or in combination with fluoxetine (FLX; 18 mg/kg/day; p.o.). Emotionality *z*-scores used in this correlation analysis concern the subpopulation of mice for which the expression of hippocampal Cx43 was examined. **(B)** Quantification of non-phosphorylated Cx43 levels in the hippocampus of mice administered with vehicle VEH or CORT given alone or in combination with FLX. **(C)** Correlation between emotionality *z*-scores and non-phosphorylated Cx43 levels in the hippocampus. Tubulin was used as a loading control. ^∗^*p* < 0.05 and ^∗∗∗^*p* < 0.001: significantly different from the VEH-treated group. ##*p* < 0.01 and ###*p* < 0.001: significantly different from the CORT/VEH-treated group. (*n* = 10–11 mice/group for *z*-scores and *n* = 5–6 for western-blot).

Because MD is multimodal, quantifiable assessment of depressive-like state is possible when different behavioral parameters can be measured in the same animal. Based on the *z*-score method, we thus normalized each parameter from the average of the corresponding values observed in the control group and integrated these values into a single score (Supplementary Table S1). We obtained an emotionality *z*-score for which the one-way ANOVA showed a significant effect of treatment factor [*F*_(2,29)_ = 32,1; *p* < 0.001]. As expected, CORT significantly increased the emotionality *z*-score relative to control animals (*p* < 0.001) and this effect was reversed by fluoxetine (*p* < 0.001; **Figure 4A**).

In a subgroup of animal subjected to the behavioral paradigms, we examined their levels of Cx43 expression in the whole hippocampus. Although one way-ANOVA revealed no effect of treatment factor on total Cx43 levels [*F*_(2,14)_ = 0,6; *p* = 0.5] (Supplementary Figure S4A), a significant effect was reported on its phosphorylated form [*F*_(2,14)_ = 5,8; *p* < 0.05]. We showed that CORT increased the levels of phosphorylated Cx43 (*p* < 0.05) and this was reversed by fluoxetine (*p* < 0.01; **Figure 4B**). Interestingly, western blot analysis did not reveal any effect of treatment factor for the expression of total Cx30 in the hippocampus [*F*_(2,14)_ = 1,2; *p* = 0.3] (Supplementary Figure S4B).

A significant positive correlation between emotionality *z*-scores and the level of expression of phosphorylated Cx43 was unveiled (Pearson *r* = 0.64; *p* < 0.01; **Figure 4C**). The higher the level of phosphorylated Cx43, the higher the emotionality *z*-score.

These results demonstrate that the anxiety and depressive-like symptoms induced by CORT correlate with an increase in the level of Cx43 phosphorylation in the hippocampus whereas chronic administration with fluoxetine normalizes this parameter.

## Discussion

In the present study, we provide evidence that the constitutive knock-down of astroglial Cx43 in mice leads to behavioral changes reminiscent of antidepressant-/anxiolytic-like activities specifically in paradigms producing a high level of stress. In parallel of these experiments and in a mouse model of depression, we also report that the antidepressant-/anxiolytic-like activities of the SSRI fluoxetine is associated with a decrease in the phosphorylation level of Cx43 in the hippocampus.

To examine the role of Cx43 on emotion-related behaviors, our initial strategy was to selectively drive the inactivation of Cx43 within hippocampal astrocytes through a local injection of pseudotyped lentivirus containing the Cre-recombinase (Colin et al., 2009). However, the observation that the expression of Cx43 in Cx43^fl/fl^ mice was dramatically decreased led us to abandon this strategy and challenge the relevance of comparing these floxed mice with their counterparts injected with the Cre-recombinase in discrete brain regions. Although the reason of such a constitutive deletion in Cx43^fl/fl^ mice remains unknown, we decided to compare the behavior of Cx43^fl/fl^ mice having a constitutive decrease in Cx43 expression as previously reported (Theis et al., 2003; Unger et al., 2012) with Cx43^wt/wt^ mice. Our results demonstrate that Cx43^fl/fl^ mice displayed an antidepressant- and anxiolytic-like phenotype in the TST and the NSF but not in the ST, the elevated plus maze and the OF. Hence, we unveiled that the constitutive Cx43 deletion improves mood and decreases anxiety specifically in paradigms producing a high level of stress. Although the role of Cx43 on these behaviors has been poorly investigated, our results are in line with earlier experiments showing increased exploratory and anxiolytic-like behaviors in mice with astrocyte-directed deletion of Cx43 in the whole brain (i.e., Cx43^fl/fl^:GFAP-Cre mice; Frisch et al., 2003). We also examined the mnesic performances of Cx43^fl/fl^ mice because learning and memory impairments represent other symptoms usually affected in MD as shown in clinical (Trivedi and Greer, 2014) and preclinical studies (Darcet et al., 2014). Interestingly, we observed that the constitutive Cx43 deletion improves the long-term memory in the contextual fear conditioning as antidepressant drugs do (for review see Keefe et al., 2014). Again, Cx43 deletion produced beneficial effects in a paradigm for which the intensity of the experienced stress is high whereas it failed to improve memory in the object location and ORTs. Together these data shed new light on the fact that deficiency in Cx43 would favor stress response whereas part of antidepressant activity might rely on the blockade of this protein. This statement is in line with a recent work showing that Cx43 in astrocytes may participate in the pathogenesis of stress-associated psychiatric disorders (Orellana et al., 2015).

To further explore the putative link between stress response and Cx43, we subjected wild-type mice to CORT and evaluated the levels of Cx expression. The chronic administration of CORT in the drinking water is a well-recognized neuroendocrine-based model of depressive-like behavior (David et al., 2009). As expected and previously reported in mice (David et al., 2009; Hache et al., 2012; Quesseveur et al., 2013b; Le Dantec et al., 2014), we showed that CORT elicited behavioral anomalies typically observed in depressed patients such as carelessness, despair, and anxiety. Because full quantifiable assessment of mood-related behavior is possible when the same animal is exposed to multiple tests covering a wide range of representative symptoms of depression, we established an emotionality *z*-score integrating all these behavioral parameters into a single value. We provided evidence that CORT significantly increased emotionality *z*-score compared to control animals as a valid index of pathological state. Indeed, the higher the emotionality *z*-score, the higher the behavioral impairments (Guilloux et al., 2011; Petit et al., 2014). On the contrary, the sustained administration of the SSRI decreased this emotionality *z*-score notably due to its ability to produce antidepressant-/anxiolytic-like activities in the ST, TST, and the NSF. Interestingly, neither CORT nor fluoxetine changed the expression of total Cx43 in the hippocampus but both treatments affected its levels of phosphorylation. One of the most remarkable result obtained herein is the fact that emotionality *z*-score is positively correlated with the levels of Cx43 phosphorylation in the hippocampus. If we consider that phosphorylation is a prerequisite for acute function of Cx (Solan and Lampe, 2009), then our results suggest that the therapeutic effects of antidepressant drugs might implicate the functional inactivation of Cx43. There is now evidence that SSRIs inhibit 5-HT uptake through the blockade of neuronal and astrocytic SERT. The subsequent enhancement of extracellular 5-HT levels likely contributes to activate astroglial 5-HT receptors (Quesseveur et al., 2013a) which, in turn, might directly regulates Cx function and/or expression. In keeping with this hypothesis, it has been shown that the application of 5-HT on hippocampal primary culture of astrocytes decreases intracellular Ca^2+^ wave propagation or dye transfer between neighboring cells (Blomstrand et al., 1999). The latter findings strengthen the hypothesis that the therapeutic activity of SSRIs would result, at least in part, from the reduction of glial cells coupling. However, these considerations are not necessarily consistent with data showing that chronic treatments with antidepressant drugs such as fluoxetine or duloxetine increase, on the contrary, astrocytic Cx43 GJC coupling (Sun et al., 2012) raising the possibility that the enhancement of astrocyte–astrocyte communication, notably in the cortex, plays an important role in antidepressant drugs response. Several explanations might be advanced to explain the discrepancies between the latter results and the findings described in the present study. The brain region (i.e., the PFC vs. the hippocampus) is an important concern that should be taken into consideration. Functional imaging studies of depressed patients indicate that the clinical response of fluoxetine is associated with a decreased functional activity of limbic regions including the hippocampus and an increased activity of the cortex (Mayberg et al., 2000). It is therefore possible that cortical and hippocampal astrocytes, according to their cellular and molecular environment, differentially impact neuronal activity. If the activation of Cx43 GJCs in the cortex seems to exert beneficial antidepressant-like effects, it is possible that its inactivation in the hippocampus is also a necessary condition to obtain similar therapeutic responses. To test for this hypothesis, we directly injected the Cx inhibitor CBX in the ventral hippocampus. However, our results indicate that the intra-hippocampal CBX elicited depressive-like state in the TST (Supplementary Figure S5) similarly to the observation made after its intra-cortical infusion (Sun et al., 2012). The latter results strongly suggest that Cx43 GJC and HC exert different effects on stress and antidepressant drugs response.

### Opposite Effects of Gap-Junctions and Hemi-Channels in Response to Stress and Antidepressant Drugs

On this background, the question may then arise as to whether the apparent beneficial effects of Cx43 down-regulation and/or inactivation on antidepressant-/anxiolytic-like activities and cognitive performances reported herein result from change in GJC and/or HC activity? The behavioral phenotyping of Cx43-deficient mice along with pharmacological studies using Cx blockers in relevant animal models of depression emphasize the importance of these proteins in the regulation of mood-related behaviors, while the respective contribution of each function is at present unknown, in particular for the HC function of Cx43.

Our results unveil a deleterious effect of Cx43 on antidepressant response. Besides their role in GJC, Cx43 also form HC to allow a direct communication with neurons by releasing gliotransmitters or other neuroactive substances in the extracellular compartment (Saez et al., 2005; Ransom and Giaume, 2013). Interestingly, acute restraint stress stimulates the opening of astrocytic Cx43 HC and a corollary of such functional change is an enhancement of glutamate release from astrocytes HCs thereby leading to neurotoxic effects (Orellana et al., 2015). These findings along with the observation that CRF stimulate the expression of Cx43 (Hanstein et al., 2009), suggest that enhanced HC activity in astrocytes could contribute to dysfunction in emotional brain circuits. In our experimental conditions, one would assume that chronic CORT exposure stimulated Cx43 HC activity to promote a prolonged and exaggerated release of glutamate in the hippocampus thereby producing neuronal damage and/or alteration in mood-related neurotransmission as previously reported in this model (David et al., 2009; Rainer et al., 2012). In this case, fluoxetine would have exerted its beneficial effects by attenuating Cx43 HC activity reflected herein by its ability to decreased Cx43 phosphorylation in response to stress. In this case, it is, however, unclear why the majority of gliotransmitters (ATP, adenosine, lactate or D-Serine) released by Cx43 are known to elicit antidepressant-like effects (for review Etievant et al., 2013) or to improve fear learning and memory in rodents (Suzuki et al., 2011; Stehberg et al., 2012; Yang et al., 2014). One explanation would be that stress specifically triggers the release of deleterious gliotransmitters in a Cx43 HC-dependent manner. Different ionic selectivities for Cx have already been observed (Wang and Veenstra, 1997) but data on gliotransmitters are still lacking. It is, however, noteworthy that the degree of Cx43 phosphorylation would be an important process to regulate such selectivity (Bao et al., 2007). Finally, we should take into consideration that Cx43 might also allow the secretion of pro-inflammatory factors such as interleukines, cytokines or chemokines. This has been already observed in response to different pathological conditions including neuropathic pain (Chen et al., 2012, 2014) and this would reconcile our results with the inflammatory hypothesis of depression.

The second hypothesis regarding Cx43 and emotionality implies that a normal activity of Cx43-mediated GJC function is required for antidepressant response. Astroglial GJC are permeable for several endogenous molecules, i.e., second messengers [cyclic AMP, inositol-1,4,5-trisphos-phate (InsP3), and Ca^2+^], amino acids (glutamate, aspartate, and taurine), nucleotides (ADP, ATP, CTP, and NAD) but also energy metabolites and their transfer facilitate information processing and integration from a large number of neurons, in providing metabolites during high neuronal demand (Giaume and Theis, 2010). Considering that antidepressant-like effects of SSRIs are associated with an enhancement of brain plasticity (cell proliferation, neuronal growth and sprouting, dendritic remodeling; Duman et al., 1999), one would expect that the facilitation of astrocyte–astrocyte communication would be in favor of a therapeutic response. As an example of the physiological importance of communication among these glial cells, we recently demonstrated that astrocytes are able to release Brain-Derived Neurotrophic Factor (BDNF) in response to fluoxetine (Quesseveur et al., 2013b). Given that such a release can be regulated by the intracellular astrocytic concentration of Ca^2+^ (Zafra et al., 1992) or glutamate (Jean et al., 2008) through Cx43 GJC, we can fairly anticipate their beneficial effects on antidepressant drug response notably owing to their proximity with neuronal synapses (Araque et al., 1999; Perea and Araque, 2010). Indeed, this widespread transfer of signaling molecules through Cx43 GJC should stimulate brain plasticity as it represents a neurotrophic supply to neurons. Interestingly, in a recent study exploring the role of astroglial Cx on various stages of hippocampal adult neurogenesis, it was reported that ablation of astroglial Cx43 significantly attenuated proliferation and reduced the survival of adult-born cells (Kunze et al., 2009; Li et al., 2010; Liebmann et al., 2013) confirming that these proteins are required to promote hippocampal adult neurogenesis and related antidepressant-/anxiolytic-like activities.

## Conclusion

Although the influence of antidepressant drugs on Cx43 expression is well described, little is known about the effects of these treatments on the functional status of Cx43 GJC and Cx43 HC. Our discussion illustrates the fact that both GJC and HC may have different effects on antidepressant drugs response leading to a complex regulation of emotionality by astrocytes. In the present study, we focused our attention on the hippocampus suggesting that the inactivation of Cx43 HC might induce beneficial effects through an attenuation of the stress response. The recent identification of anatomical and functional interactions between the hippocampus and the hypothalamus (Radley and Sawchenko, 2011) reinforces the interest to further explore the influence of hippocampal astroglial Cx43 in the regulation of the neuroendocrine system.

## Author Contributions

GQ in charge of the behavioral experiments in mice subjected to corticosterone. BP western-blot analysis in Cx43^fl/fl^ mice. J-AB in charge of the behavioral experiments in Cx43^fl/fl^ mice. PE western-blot analysis in mice subjected to corticosterone. AM in charge of behavioral experiments. HH mice genotyping. CL article writing. Scientific expertise. XF in charge of the behavioral experiments in Cx43^fl/fl^ mice. ND article writing. Scientific expertise. CG provide de Cx43^fl/fl^ mice. Article writing. Scientific expertise. CR article writing. Scientific expertise. BG project designer – article writing.

## Conflict of Interest Statement

The authors declare that the research was conducted in the absence of any commercial or financial relationships that could be construed as a potential conflict of interest.

## Supplementary material

FIGURE S1 **Schematic representation of the experimental designs. (A)** Cx43^wt/wt^ and Cx43^fl/fl^ mice were subjected to behavioral tests over a 1-week period. **(B)** Wild-type mice were subjected to a 8-week exposure to corticosterone (CORT) followed by a 4-week exposure to fluoxetine or its vehicle. Behavioral tests were then conducted for 1 week with a 2-day period between each test.

FIGURE S2 **(A)** Survival curve showing the fraction of animal not eating during the novelty suppressed feeding (NSF). **(B)** Similar body weight loss (in gr) between Cx43^wt/wt^ and Cx43^fl/fl^ mice after a 24-h period of fasting. **(C)** Similar home cage consumption (in gr/gr of mouse body weight) during 5 min between Cx43^wt/wt^ and Cx43^fl/fl^ mice after the NSF test. **(D,E)** Time of exploration of the objects in the OL **(D)** and OR **(E)** during the acquisition phas at D1. **(F)** Time of freezing before the shock in the FCF.

FIGURE S3 **(A)** Survival curve showing the fraction of animal not eating during the NSF. **(B)** Similar body weight loss (in gr) between VEH/VEH-, CORT/VEH-, and CORT/FLX-treated mice after a 24-h period of fasting. **(C)** Similar home cage consumption (in gr/gr of mouse body weight) during 5 min between VEH/VEH-, CORT/VEH-, and CORT/FLX-treated mice after the NSF test.

FIGURE S4 **Expression of total astroglial Cxs. (A)** Levels of Cx43 in VEH/VEH-, CORT/VEH-, and CORT/FLX-treated mice. **(B)** Levels of Cx30 in VEH/VEH-, CORT/VEH-, and CORT/FLX-treated mice.

FIGURE S5 **Depressive-like effect of intra-hippocampal infusion of an astroglial Cx43 blocker in wild-type mice in the tail suspension test. (A)** Experimental protocol. Wild-type mice were bilaterally implanted with cannula within the ventral hippocampus and after a 1-week period of recovery, they were tested in the tail suspension test 15 min after the intra-hippocampal infusion of carbenoxolone (CBX 10 mM) or its vehicle. The same animals were tested 1 week later with a higher dose of CBX (100 mM). **(A,B)** Data are expressed as mean ± SEM of the time of immobility after CBX (10 mM) **(B)** or (100 mM) **(C)**. **p* < 0.05: significantly different from the VEH-infused group (*n* = 7 mice/group).

TABLE S1 **Mathematical method for Emotionality score calculation.** The *Z*-score is a dimensionless mathematical value obtained by subtracting each individual data (X) from the mean of the control group (**μ**) and then dividing the difference by the standard deviation of the control group (**σ)**. *Z*-score of the Splash Test (ST), the Elevated Plus Maze (EPM) and the Open Field (OF) were converted into opposite values (i.e., by putting the negative sign in front of the formula) in order to standardize the directionality of these scores and to ensure that increased values reflected a pathological state. An individual *z*-score was therefore determined for each parameter. In the case of behavioral tests integrating multiple parameters, such as the EPM and the OF, an averaged *Z*-score was calculated across parameters. Locomotor activity in the EPM and OF was integrated in the averaged *Z*-score of these tests in order to avoid potential biases induced by locomotion on anxiety-like behaviors. Finally, an individual Emotionality score is obtained by averaging *Z*-score of all tests.
