# Supplementary figures and images for: Attenuated Levels of Hippocampal Connexin 43 and its Phosphorylation Correlate with Antidepressant- and Anxiolytic-Like Activities in Mice
Source: Front Cell Neurosci. 2015 Dec 22;9:490. doi: 10.3389/fncel.2015.00490 (PMC4686612; doi:10.3389/fncel.2015.00490)

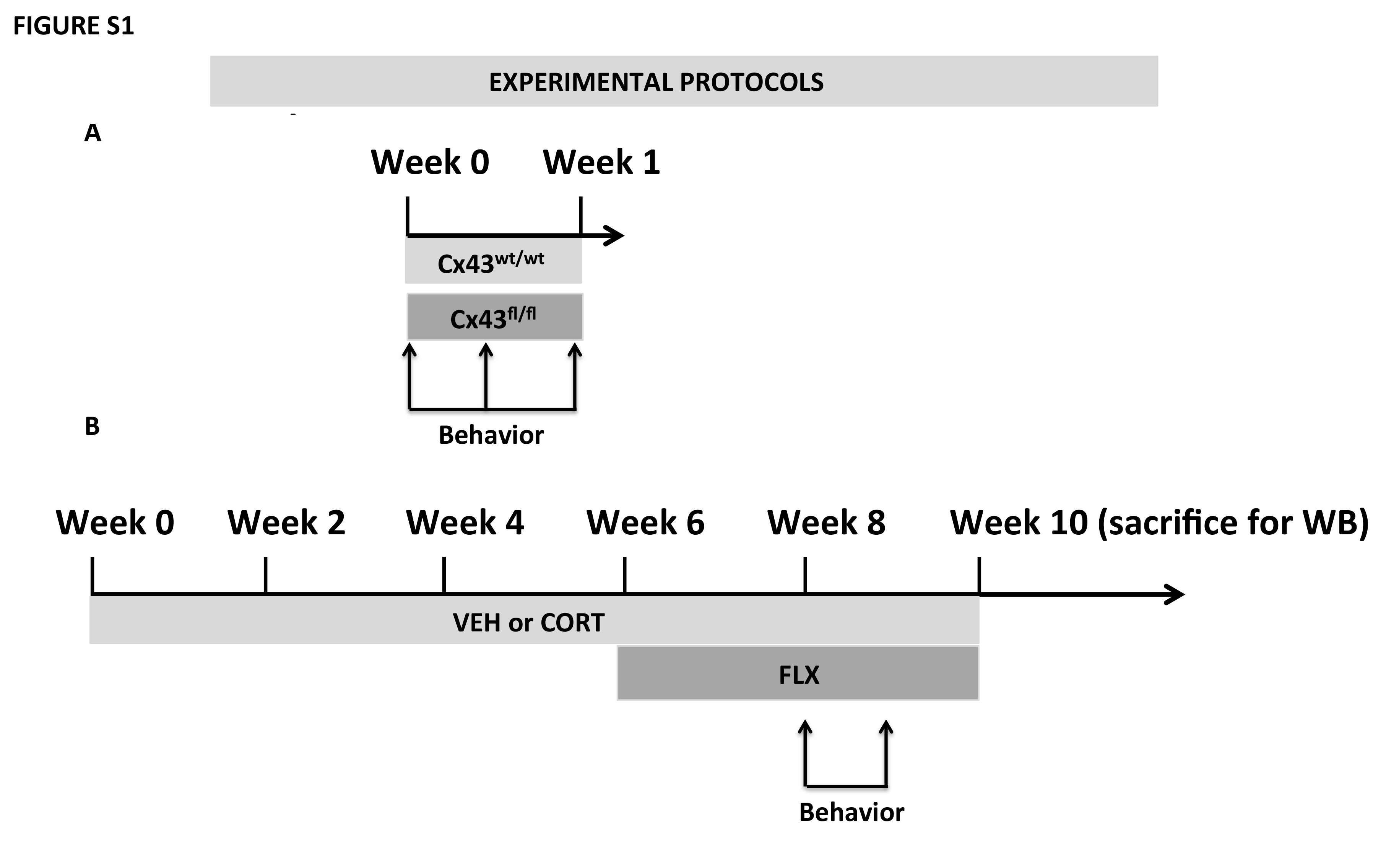

Supplement: FIGURE S1 — Schematic representation of the experimental designs. (A) Cx43wt/wt and Cx43fl/fl mice were subjected to behavioral tests over a 1-week period. (B) Wild-type mice were subjected to a 8-week exposure to corticosterone (CORT) followed by a 4-week exposure to fluoxetine or its vehicle. Behavioral tests were then conducted for 1 week with a 2-day period between each test. [file Image_1.JPEG]

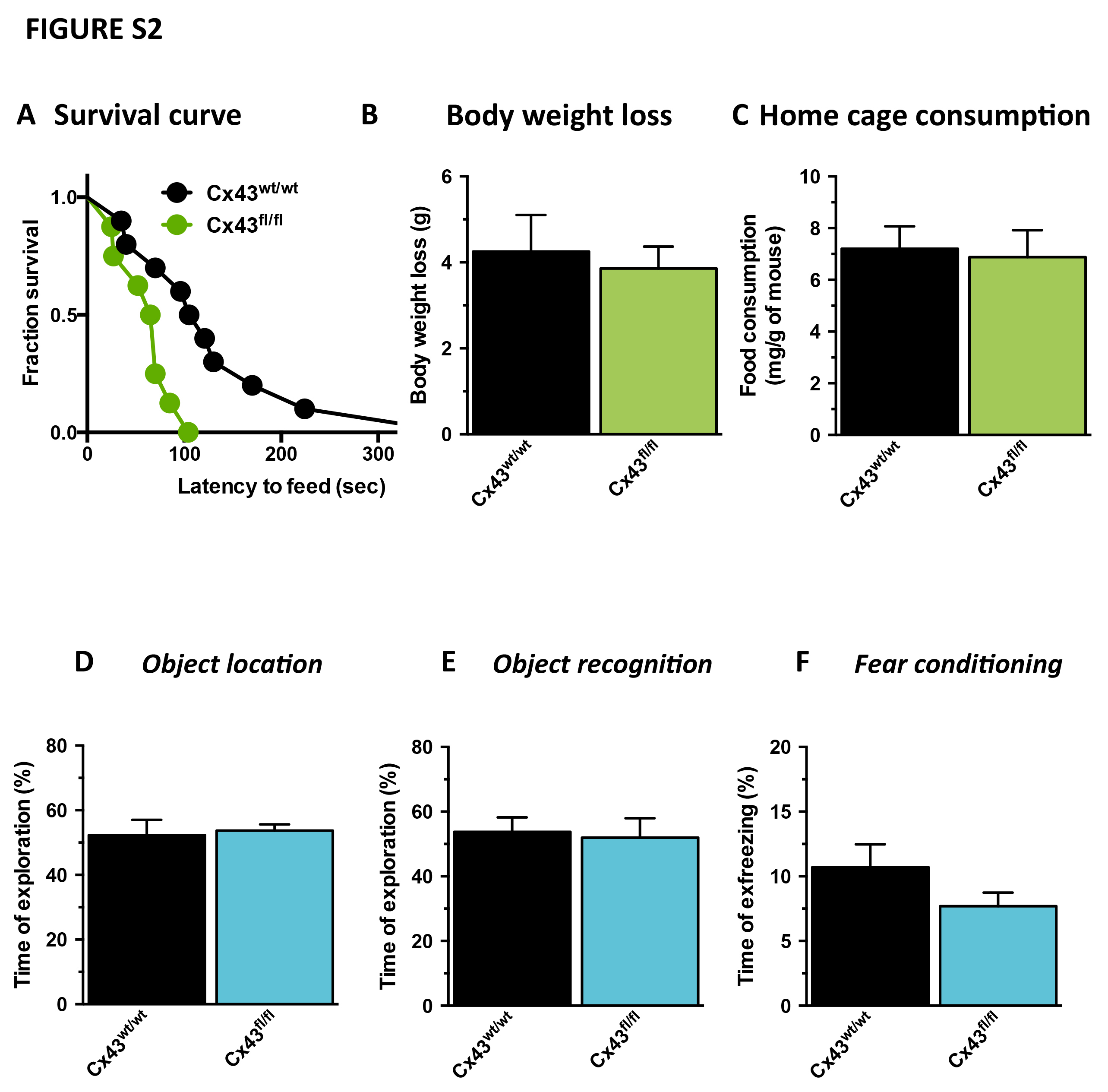

Supplement: FIGURE S2 — (A) Survival curve showing the fraction of animal not eating during the novelty suppressed feeding (NSF). (B) Similar body weight loss (in gr) between Cx43wt/wt and Cx43fl/fl mice after a 24-h period of fasting. (C) Similar home cage consumption (in gr/gr of mouse body weight) during 5 min between Cx43wt/wt and Cx43fl/fl mice after the NSF test. (D,E) Time of exploration of the objects in the OL (D) and OR (E) during the acquisition phas at D1. (F) Time of freezing before the shock in the FCF. [file Image_2.JPEG]

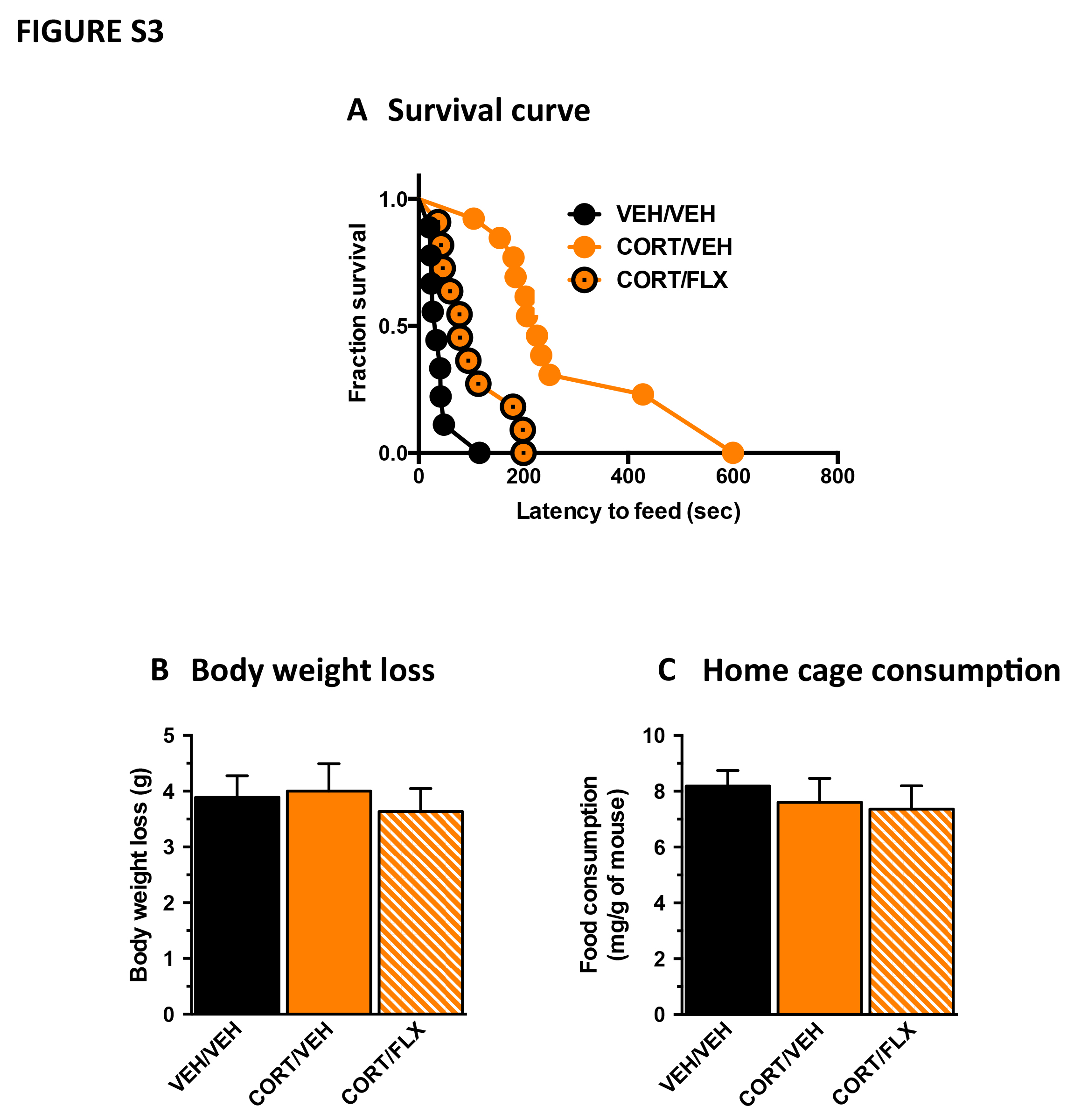

Supplement: FIGURE S3 — (A) Survival curve showing the fraction of animal not eating during the NSF. (B) Similar body weight loss (in gr) between VEH/VEH-, CORT/VEH-, and CORT/FLX-treated mice after a 24-h period of fasting. (C) Similar home cage consumption (in gr/gr of mouse body weight) during 5 min between VEH/VEH-, CORT/VEH-, and CORT/FLX-treated mice after the NSF test. [file Image_3.JPEG]

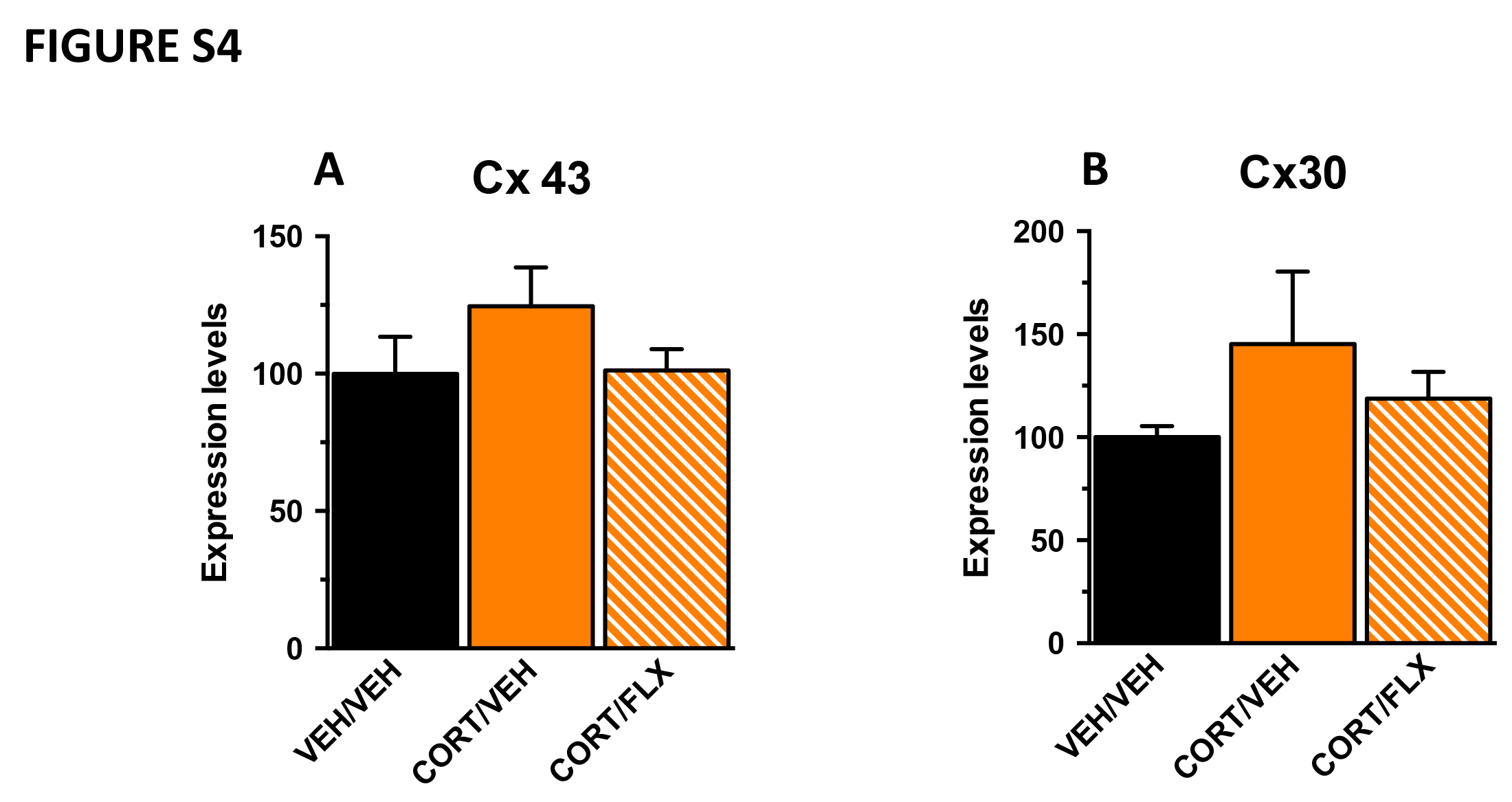

Supplement: FIGURE S4 — Expression of total astroglial Cxs. (A) Levels of Cx43 in VEH/VEH-, CORT/VEH-, and CORT/FLX-treated mice. (B) Levels of Cx30 in VEH/VEH-, CORT/VEH-, and CORT/FLX-treated mice. [file Image_4.JPEG]

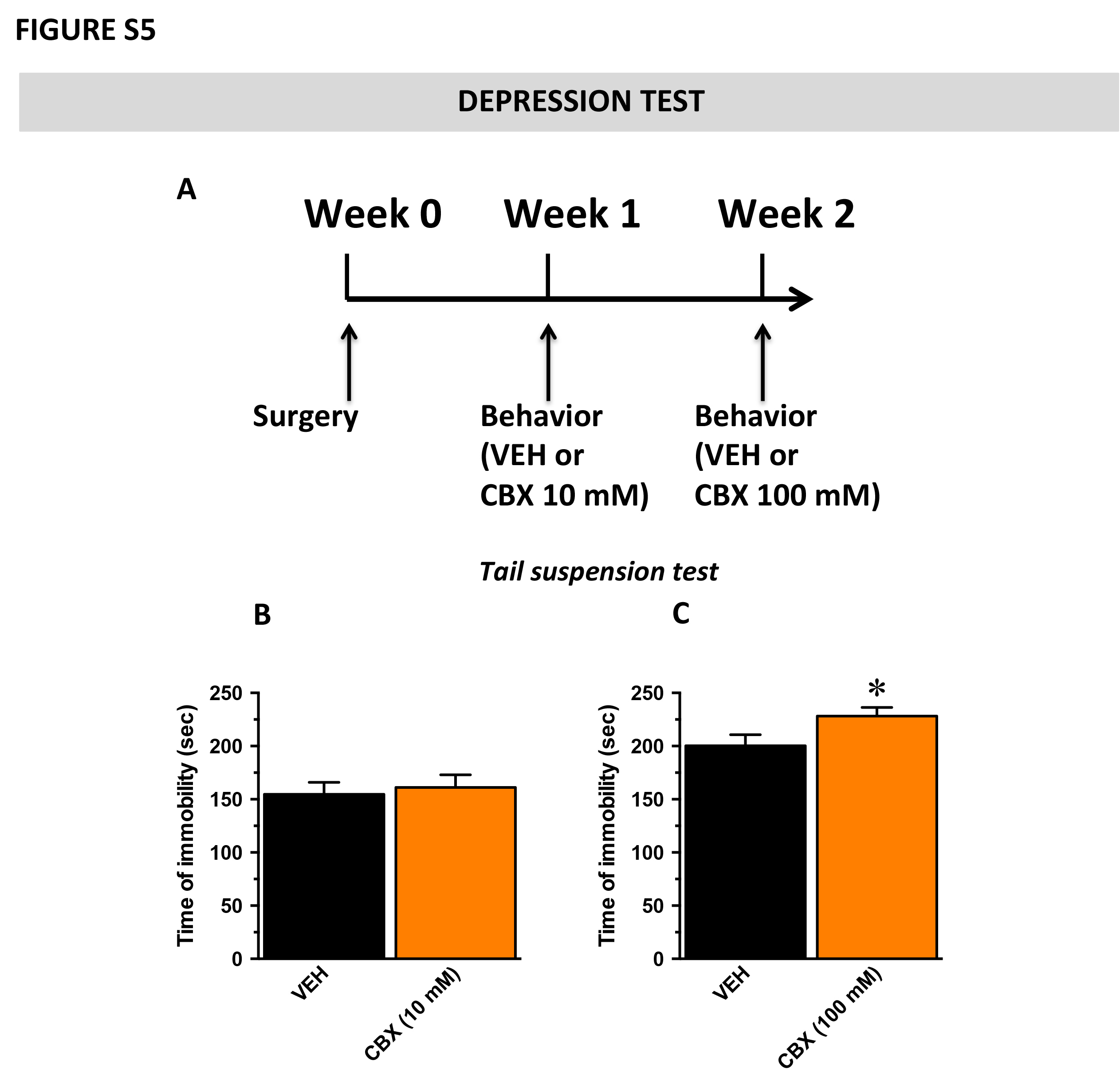

Supplement: FIGURE S5 — Depressive-like effect of intra-hippocampal infusion of an astroglial Cx43 blocker in wild-type mice in the tail suspension test. (A) Experimental protocol. Wild-type mice were bilaterally implanted with cannula within the ventral hippocampus and after a 1-week period of recovery, they were tested in the tail suspension test 15 min after the intra-hippocampal infusion of carbenoxolone (CBX 10 mM) or its vehicle. The same animals were tested 1 week later with a higher dose of CBX (100 mM). (A,B) Data are expressed as mean ± SEM of the time of immobility after CBX (10 mM) (B) or (100 mM) (C). *p < 0.05: significantly different from the VEH-infused group (n = 7 mice/group). [file Image_5.JPEG]
