# Supplementary material for: Attenuated Levels of Hippocampal Connexin 43 and its Phosphorylation Correlate with Antidepressant- and Anxiolytic-Like Activities in Mice
Source: Front Cell Neurosci. 2015 Dec 22;9:490. doi: 10.3389/fncel.2015.00490 (PMC4686612; doi:10.3389/fncel.2015.00490)
Supplement: TABLE S1 — Mathematical method for Emotionality score calculation. The Z-score is a dimensionless mathematical value obtained by subtracting each individual data (X) from the mean of the control group (μ) and then dividing the difference by the standard deviation of the control group (σ). Z-score of the Splash Test (ST), the Elevated Plus Maze (EPM) and the Open Field (OF) were converted into opposite values (i.e., by putting the negative sign in front of the formula) in order to standardize the directionality of these scores and to ensure that increased values reflected a pathological state. An individual z-score was therefore determined for each parameter. In the case of behavioral tests integrating multiple parameters, such as the EPM and the OF, an averaged Z-score was calculated across parameters. Locomotor activity in the EPM and OF was integrated in the averaged Z-score of these tests in order to avoid potential biases induced by locomotion on anxiety-like behaviors. Finally, an individual Emotionality score is obtained by averaging Z-score of all tests. [file Table_1.DOCX]

**X** : Individual data for the observed parameter ; **μ** : Mean of the control group ;

**σ** : Standard deviation of the control group
